# Practical Departments

**Published:** 1906-04-07

**Authors:** 


### PRACTICAL DEPARTMENTS.

### MECHANICAL AND OTHER BEDSTEADS.

Messrs. Chorlton, of Blackfriars Street, Manchester, have applied themselves to the task of supplying every kind of bedstead for use in the sick-room and in institutions for the care of the sick. They have a special cross-tension spring mesh which can be fitted to any bedstead, which is very superior from the important point of durability. The patent bedsteads for epileptics are ingeniously supplied with every possible protection for the patient. Peter's patent turnover bedstead removes the difficulty of turning a helpless patient by hand. The "Dual Bottom" design and "Surgical" bedsteads both are excellent for special cases. Turn-up folding bedsteads, sanatorium chairs and couches, bed-rests, and tabes form a few of the most notable items in a catalogue which cannot fail to interest those connected with the administration of hospitals, etc. Ask for catalogue O.

